# Supplementary material for: Are carbon emissions trading and green financial instruments synergistic? -Comprehensive quantitative research based on content analysis
Source: PLoS One. 2024 Mar 7;19(3):e0298601. doi: 10.1371/journal.pone.0298601 (PMC10919601; doi:10.1371/journal.pone.0298601)
Supplement: S1 Data — (ZIP) [file pone.0298601.s001.zip › Data/地级市Z_F工作报告.zip!/╔╜╢1⁄2/╝├─╧╩╨/└┤╫╘╣1⁄2╓┌║┼í╛┬φ┐╦╩2╛▌═°í┐_2014.docx]

各位代表：

　　现在，我代表市人民政府向大会报告工作，请予审议，并请各位政协委员和其他列席人员提出意见。

一、2013年工作回顾

　　2013年，是我们深入贯彻落实党的十八大精神、积极应对错综复杂经济形势、加快省会转型发展的关键一年。在中共济南市委坚强领导下，市政府紧紧围绕“加快科学发展，建设美丽泉城”中心任务，按照稳中求进的工作总基调，凝心聚力，攻坚克难，全力做好稳增长、调结构、促改革、惠民生各项工作，经济社会发展稳中有进、稳中向好，完成了十五届人大二次会议提出的主要目标任务，省会现代化建设取得新的进展。

　　（一）在稳定经济增长中提升发展质量效益。面临经济下行的压力，我们加快转调创步伐，扩大有效需求，狠抓招商引资、项目建设和实体经济，实施系列创新举措，积极作为，扎实推进，经济运行稳中有进、逐季向好，质量效益有了新的提高。预计全年实现生产总值5200亿元以上，增长9.6%左右；地方公共财政收入482.1亿元，可比口径增长13.9%；其中地方税收收入381.9亿元，增长15.1%；税收比重达到79.2%，提高3.2个百分点；规模以上工业利税、利润分别增长25%和42%；社会消费品零售总额增长13%左右。结构调整积极推进。服务业占生产总值比重提高到55%，高新技术产业产值占全市规模以上工业比重为40.55%，分别提高0.6和1个百分点。重汽、齐鲁制药等一批企业主动调整产品结构、开拓新兴市场，实现销售和效益双增长，二机床集团竞得福特美国工厂第6条大型智能冲压生产线订单，开启“济南创造”进军国际市场新征程；天岳碳化硅、华云豪克能、建邦新材料等一批新兴高科技企业崭露头角；电子商务交易额突破千亿元，软件和信息服务业收入增长25%。发展后劲逐渐增强。完成固定资产投资2600亿元、增长21%，是近5年来最高增幅；战略性新兴产业投资增长25%；扎实推进220个重点项目建设，建成了华芯富创OGS触控屏、齐鲁电机、圣泉20万吨酚醛树脂等一批投入大、带动力强、附加值高的好项目；创新谷、济南药谷、汉峪金谷等一批孵化器、平台加快建设，吸引了大批企业集聚发展。宝世达、汇丰铸造、玫德铸造3家企业获批国家级企业技术中心。各类市场主体新增1.7万户、增长25.4%。山东企业上市路演中心建成投用，全市金融机构本外币存款余额突破万亿元。全面完成淘汰落后产能和节能减排任务。

　　（二）在拓展发展空间中提升城市功能品质。我们按照建设美丽泉城总体要求，以片区开发、重大基础设施建设为支撑，进一步拓展空间、增强功能、彰显特色、提升内涵。编撰《济南市三年建设规划》，推出了仲宫、柳埠、西营三镇控制性详规、南部山区旅游规划、北部新城战略规划等一批专项规划和产业规划。从完善规划、政策推动、项目建设、产业发展等方面，积极推进北跨发展，黄河以北地区承载发展能力进一步增强。兴隆、南北康、华山、雪山、非物质文化遗产博览园等一批片区拆迁建设加快推进，千佛山风景区、泉城特色标志区、火车站北场站一体化等规划建设取得积极进展，省会文化艺术中心、绿地普利中心等一批文化、商业新地标拔地而起，省会大剧院入选2013年中国十大剧院，西部新区道路框架基本形成，绽露现代化都市新形象。一批重大交通设施建设加快推进，城市轨道交通建设启动，济南机场南指廊工程即将建成，济南至东营、济南至乐陵高速公路加紧施工，二环西路高架北段建成通车，二环西路南延、二环南路高架动工建设，泉城路、旅游北路、土屋路等一批主次干道整治完工。围绕保障新区开发和老城提升，配套完成一批供水、供热、供气、供电、公交和生活垃圾处理等市政工程，南水北调东线一期正式通水。扎实开展水生态文明市创建等“五城联创”工作，完成兴济河、工商河、巨野河等一批河道治理工程和30条小流域综合治理，敷设雨污管线170余公里，基本实现城区河道污水不直排。深入推进市容道路、铁路沿线、建筑渣土、违法建设等11项整治工作，继续实施城市交通综合整治三年行动计划，改善市区交通拥堵状况。综合施策整治雾霾天气，推进燃煤锅炉“煤改气”和扬尘、渣土治理，在建成区禁行黄标车。突出城市出入口、重要道路及节点绿化美化，森林覆盖率达到34%、提高1.7个百分点，城市绿化更加丰富多彩。采取增雨置采、水源连通等措施，实现趵突泉群连续十年喷涌。经过不懈努力，天下第一泉风景区荣膺国家5A级旅游景区，结束了我市没有5A级景区的历史。采取市场化运作模式，成功举办济南首届泉水节，圆了泉城市民多年的梦想。

　　（三）在促进协调发展中激发县（市）区经济活力。为充分发挥县（市）区发展主体作用，市委、市政府在实施三年突破平阴、推动长清加快融入中心城区发展的基础上，出台支持济阳加快建设北跨产业发展区、继续支持商河跨越发展等政策措施，在基础设施建设、产业发展、招商引资、财源建设等方面，赋予县区、园区更多的发展决策权和管理权，为县区经济发展注入新动力。各县（市）区抢抓政策机遇，不断调整、丰富、完善发展思路，发挥优势，创新突破，进一步增强内生动力，呈现各具特色、良性互动的发展新格局。在2012年济阳县财政收入过10亿元基础上，又有平阴、长清两个县（区）突破10亿元，历下区突破百亿元，其它县（市）区财政收入均有大幅增加。中心城区服务业发展有了新提升，以片区改造为载体，培育税源型产业，中海环宇城建成运营，鲁商国奥城、祥泰广场、领秀城、银座好望角等一批高端商务楼宇加快建设，引进东亚银行、安顾保险等地区总部，济南金融商务中心区、黄金珠宝交易中心建设取得积极进展。周边县域依托明水经济开发区、济北经济开发区等园区，加快工业发展和项目建设，支持福胶、宏达、元首、大汉等本土企业扩规发展，历城现代物流、长清压力容器、章丘精细化工、平阴炭素、济阳食品、商河纺织等一批中小产业集群快速崛起，成为县域经济增长的新引擎。以崔寨、刁镇、玉皇庙等省级示范镇为引领的镇域发展有了新起色，加大县域基础设施建设，城乡生活垃圾处理覆盖率达到95%，城乡面貌明显改观。农业经济实现新发展，粮食生产实现十一连丰。农业产业化进程加快，农业现代经营服务体系不断完善，涌现出一批种植、畜牧、水产养殖家庭农场，农业龙头企业、农民合作社分别发展到473家、4808家。推进农高区、章丘现代农业示范园等“一区六园”建设，都市农业品牌特色基地加快发展，新建高标准基本农田51万亩，整理复垦土地18万亩、新增耕地2.2万亩。实现6.5万贫困人口人均增收1428元。

　　（四）在深化改革开放中不断优化体制环境。我们按照市委、市政府深化改革扩大开放总体部署，扎实推进行政审批制度、科技创新体系等十项重点领域改革，出台调整财政管理机制、重大项目绿色审批、节约集约用地、重点项目用地指标竞争分配、提升行政效能等系列措施办法，进一步简政放权，创新体制机制，释放发展活力，为我市全面深化改革打下良好基础。一年来，先后对42个部门168项行政许可、223项行政责任事项进行梳理审查，进一步压缩审批时限，向县（市）区下放审批权限166项，推行“一号通”、“一表通”审批新模式。创新财政资金使用方式，整合部分产业引导资金，变财政拨款为股权投资，引导社会资金投向实体经济、科技创新和文化产业领域。扎实做好“营改增”试点工作，落实各项税收减免政策，为企业减免税收累计157.2亿元。稳妥推进城镇化试点，探索户籍制度改革，实施土地承包经营权确权登记颁证试点，城镇化率达到66%左右。创新招商引资机制方式，开展聚焦招商、载体招商、产业链招商，组织赴北京、香港、台湾、韩国及东南亚、欧洲等招商活动，成功举办第七届华交会，促进了一批大项目签约落地，形成台湾工业园、欧洲工业园等外资企业集聚发展的格局。综合保税区通过封关验收，航空城规划建设加快推进。全市实际到账外资增长8%，进出口增长5%。对外友好交往有新进展，增加1个友好城市、5个友好合作城市。贯彻落实省会城市群经济圈发展战略，组织召开第一次党政联席会议，建立健全合作机制，成立济莱协作区工作推进机构，协同各城市强化产业配套、要素配置以及道路交通等重大基础设施建设，促进区域紧密型合作、一体化发展。

　　（五）在强化为民办实事中提升公共服务水平。我们坚持增加民生投入与加大工作力度并举，集中力量解决了一批民生问题，提升了公共服务和社会管理水平。民生和重点社会事业支出占财政支出的比重达到58.6%。全面完成为民办17件实事。城市居民人均可支配收入、农民人均纯收入分别增长9.5%和11%左右，居民消费价格涨幅为2.8%左右。把就业作为民生工作的头等大事，通过加强就业扶持、培训提升、创业指导，新增城镇就业18.55万人，农村劳动力转移就业7.3万人。建立市、区两级农民工综合服务中心，提供职业介绍、教育培训、权益维护、社会保障等一揽子服务，帮助农民工更好地融入城市。各项社会保险参保人数和基金收入创历史新高，企业退休人员月人均养老金达到2171.5元，城乡居民基本养老、医疗待遇水平进一步提高。推进教育资源均衡配置，稳妥实施中考制度改革，全面完成中小学校舍安全工程五年规划任务和普通中小学标准化建设工程，基本完成学前教育三年行动计划，新增16所外来务工人员子女学校。县级公立医院综合改革扎实推进，“新农合”人均筹资标准达到350元。加快农村住房建设与危房改造，启动农村新型社区建设项目50个、建设农房2万余户、改造危房1913户。提前超额完成保障性住房建设任务，开工建设各类保障性安居工程16370套，竣工3683套，实际配租房源8717套。启动帮扶国有困难企业和困难职工工作，向44户市属国有企业逐户派驻工作协调组，筹集5.35亿元启动资金，因企制宜，一企一策，努力使一批有条件的企业实现脱困，焕发生机活力。成功举办第十届中国艺术节，我们按照中央节俭办会的要求，扎实做好比赛场馆及公共文化设施建设，提供高效文明的服务保障和赛事组织，开展“全民共参与，十艺更精彩”和“十艺济南”等系列群众文化活动，打造了“艺术的盛会、人民的节日”，我市参评剧目《项羽》获文华大奖特别奖，《泉城人家》获文华优秀剧目奖，《幸福像花儿一样》等4件作品和“书香泉城•全民阅读节活动”等2个项目获“群星奖”。在第十二届全国运动会上我市运动员荣获28枚金牌，取得了我市竞技体育历史最好成绩。

　　（六）在开展群众路线教育实践活动中转变政府工作作风。按照中央统一部署和省委、市委安排，从去年7月中旬以来，市政府及市直各部门扎实开展党的群众路线教育实践活动，紧扣为民、务实、清廉主题，按照“照镜子、正衣冠、洗洗澡、治治病”总要求，认真学习习近平总书记一系列重要讲话和党的群众路线重要论述，深入工地、社区、农村、企业、高校院所、中央和省驻济单位、部队，广泛征求意见建议，深刻查摆形式主义、官僚主义、享乐主义和奢靡之风问题，通过边学边改、建章立制、明确重点、督促落实，解决了一批影响改革发展和群众反映强烈的突出问题。深入开展厉行勤俭节约反对铺张浪费和治理庸懒散专项活动，加强政府工作人员绩效考核，促进了政府作风转变和全面提速提效。自觉接受人大依法监督和政协民主监督，严格依法行政，认真办结人大代表建议306件、政协委员提案611件。健全反腐倡廉和科技防腐机制，构筑惩治和预防腐败体系。深入推进平安济南建设，社会治安形势稳定。文明城市创建工作取得新进展，在全国城市文明程度指数测评中，我市2012、2013两年总成绩居省会副省级提名资格城市第一名。荣膺全国首批法治城市创建先进市和首个国家防震减灾示范城市创建市。12345市民服务热线成为国家政务服务热线标准主导制定单位。侨务、对台、人口计生、民族宗教、妇女儿童、慈善、老龄、残疾人、档案史志、人民防空、双拥共建以及援藏援疆等工作取得新的进步。

　　各位代表，2013年刚刚过去，我们又迎来了全面深化改革开放的新征程。此刻，我们更加深切地感受到，过去一年的成绩，是在党中央国务院和省委省政府、市委正确领导下，全市广大干部群众团结奋斗的结果。人民群众永远是省会发展的力量源泉，是美丽泉城的建设者。在这里，我代表济南市人民政府，向全市各族人民，向给予我们不懈支持的人大代表、政协委员、各民主党派、工商联和各界人士，向驻济部队、武警官兵和中央、省驻济单位，向港澳台同胞、海外侨胞和国际友人，向在省会辛勤工作的外来务工人员，表示崇高敬意和诚挚感谢！

　　在送走忙碌而充实的一年、总结收获与进步的时刻，我们也清醒地认识到，我市经济社会发展中还存在不少矛盾和问题，发展中不平衡、不协调、不可持续的问题仍然突出，综合实力不强、发展后劲不足、产业结构不优的问题还不同程度地存在，转方式调结构任务艰巨，新的增长点培育较慢；资源环境约束加剧，节能减排压力加大，生态文明建设形势逼人；城市基础设施、综合服务功能、城市管理水平还不能满足建设美丽泉城的需要，南部山区绿色发展、北部跨河发展的基础还比较薄弱；民生工作与群众期望还有一定差距，在安全生产等方面还存在薄弱环节；体制机制环境仍然不能适应创新驱动、转型升级的迫切要求，服务发展、推动工作、狠抓落实的力度和成效需要进一步提高，整治“四风”、反对腐败任重道远。这些问题，有些是长期积累的，有些是在新形势下出现的，还有一些是政府工作中的不足造成的。我们要以对省会发展和人民群众高度负责的态度，努力用改革创新的思路、艰苦奋斗的精神、扎实高效的工作，切实加以解决。

二、2014年工作安排

　　2014年，是全面贯彻落实党的十八届三中全会精神、全面深化改革的第一年，也是“加快科学发展，建设美丽泉城”、完成“十二五”规划的攻坚之年。当前，国际经济缓慢复苏，我国经济正处于增长速度换档期、结构调整阵痛期叠加的阶段，挑战和机遇并存，特别是党的十八届三中全会对全面深化改革作出总体部署，进一步激发了转型发展的内生动力和创新活力。我市改革发展、转型升级正逢其时。我们必须立足当前、着眼长远，凝聚共识、坚定信心，始终扭住发展这个解决所有问题的关键，牢牢把握重大调整机遇，切实实现工作指导重大转变，卓有成效地把深化改革、转型升级、发挥优势、壮大实力结合起来，扎实办好自己的事情，推动省会改革发展再上新台阶。

　　今年政府工作的总体要求是：全面贯彻落实党的十八大、十八届三中全会、习近平总书记系列重要讲话精神，按照市委十届五次全会部署，坚持稳中求进工作总基调，紧紧围绕“加快科学发展，建设美丽泉城”中心任务，切实把提高发展质量和效益放在中心位置，把改革创新贯穿经济社会发展各个领域各个环节，加快转方式调结构，继续壮大实体经济，完善城市功能，提高城镇化质量，强化生态建设，着力改善民生，促进经济持续健康发展、社会和谐稳定。

　　今年经济社会发展的主要预期目标是：生产总值增长9%，地方公共财政收入增长12%左右，固定资产投资增长18%，社会消费品零售总额增长13%。农业、服务业、规模以上工业增加值分别增长4%、10%和10%，高新技术产业产值比重提高1个百分点。进出口增长6%，实际到账外资增长5%。城市居民人均可支配收入增长10%左右，农民人均纯收入增长10%以上，居民消费价格涨幅控制在3.5%左右，城镇登记失业率控制在4%以内，人口自然增长率控制在6‰以内，完成省政府下达的年度节能减排和环境保护任务。重点抓好以下工作：

　　（一）打好全面深化改革攻坚战，创新体制机制，激发市场活力，增强科学发展的内生动力

　　改革是发展的最大动力，体制机制创新是提升城市核心竞争力的关键。前不久，市委十届五次全会审议通过了《关于深入学习贯彻党的十八届三中全会精神全面深化改革的实施意见》，对全面深化改革作出了部署。我们一定要以改革统领经济社会发展全局，坚定信心，凝聚力量，更好发挥市场配置资源的决定性作用和政府的作用，积极稳妥推进重要领域和关键环节改革，落实好《实施意见》提出的46项改革举措，为经济社会发展增添新动力、开辟新空间。

　　进一步简政放权。继续清理精简行政审批事项，能取消的一律取消，该由市场做的放给市场，社会能办的交给社会，基层能管好的下放县区和园区，切实提高经济运行效率。在去年基础上，今年再取消行政审批30项、审批要件148项、行政备案事项27项、行政事业收费33项，并实行动态精简机制，推进审批服务“两集中、两到位”和建设项目限时审批。放宽准入条件，取消有限公司注册登记最低资本限制，推行“认缴登记”、“先照后证”。在高新区试点民营资本“负面清单”管理，在综合保税区试点外资“负面清单”管理，并逐步在全市推开。推进管理重心下移，加大规划、土地、建设等重点领域向基层放权力度，激发县区改革发展活力。各县（市）区也要承接好各项下放权限，确保落实到位。

　　强化服务监管职能。按照公共服务、市场监管、社会管理、环境保护的职责，全面正确履行政府职能。建立政府部门权力清单制度，重新梳理、归并、界定部门行政权责，依法公开权力运行流程，着力解决职能错位、越位和缺位问题。深化财税体制改革，构建公开透明的预算制度，强化债务风险防控，认真落实“营改增”等结构性减税政策。搞好保障民生的基本公共服务，依托市政务服务中心，健全政府购买服务机制，调动市场力量发展社会服务，提升公共服务供给水平。加强对市场全过程监管，把问题企业和违法经营者列入“黑名单”，惩治假冒伪劣、坑蒙拐骗、金融诈骗、恶意欠薪、侵犯知识产权等行为，完善社会信用体系，打造重诺守信的营商环境。

　　激发市场主体活力。深化国企改革，健全市属企业国有资产监管机制，改革国有资本授权经营，完善国有资本经营预算制度，扩大国有资本收益上缴覆盖面，加强现代企业制度建设，强化国有企业经营投资责任追究。坚持“发展、救助、改制”三管齐下，扎实做好44家国有困难企业帮扶脱困工作，统筹解困振兴、改制退出和企业职工妥善安置。大力发展非公有制经济，落实鼓励民营经济各项政策，在水、电、气、暖、垃圾处理、出租车等特许经营领域制定非公企业进入的标准和程序，努力为中小微企业创新发展提供服务保障。支持混合所有制经济发展，鼓励国有资本、集体资本、非公有制资本等交叉持股、相互融合，提升市场主体创造力和活力。

　　（二）坚持壮大实体经济，着力培育新的经济增长点，确保实现有质量的增长

　　把壮大实体经济作为保持经济持续健康发展的重要支撑，切实发挥好投资的关键作用、消费的基础作用，在优化存量基础上，着力增加消费需求，促进投资合理增长和结构优化，不断增强省会经济综合实力。

　　坚持抓项目、促招商、培育新增量。继续实施项目建设三年行动计划，按照签约项目抓开工、在建项目抓进度、竣工项目抓达效的要求，围绕新兴产业发展、传统产业振兴、基础设施建设、生态环境保护和民生改善，加快220个市级重点项目建设，强化规划、国土、环保、审批等服务保障，用好绿色通道推进机制，增加有效投入，确保投资合理增长，特别要促成11类136个实体经济重点项目尽快投产达效。围绕促进产业升级、链式发展、创新带动、税源培植，超前研究策划一批储备项目。按照“四抓四不忘”的要求，完善创新招商机制，推进产业链招商、以商招商、聚焦招商和飞地招商，切实发挥企业和各类商会的作用，强化职能部门和县（市）区招商工作推进责任。坚持引资与引智、引技相结合，引项目与引产业、引平台、引总部相结合，强化招商选资工作。进一步放宽民间资本投资领域，提高民营经济和民间投资比重。

　　坚持拓领域、扩市场、培育新增量。注重在文化旅游、电子商务、住宅产业化、节能环保、健康养老、现代农业等新产业领域，提早筹划布局，抢占市场先机，培育发展新增量。注重理顺体制、整合资源、打造品牌、拓展市场，做大文化旅游产业，加快西客站游客集散中心、非物质文化遗产博览园、龙冈梦幻星空、融汇温泉城、澄波湖风景区等项目建设，发挥旅游传播文化、集聚人气、拉动增量消费的作用。以国家电子商务示范城市建设为契机，实施电子商务引领发展战略，完善电子商务运营服务体系，推进高新区、历下区、明水经济技术开发区电子商务产业园等综合服务平台建设，培育发展一批电子商务企业，推动装备制造、商贸流通等领域电商化。扎实推进国家住宅产业化综合试点城市建设，探索通过容积率奖励、公租房启动试点等政策措施，促进住宅产业化规模发展。围绕节能减排潜力大、市场需求明显的重点领域，加快技术研发、推广和产业化，带动节能环保产业全面提升。依托省会丰富的医疗和服务资源，打造一批辐射全省的养老、健康服务企业和产业基地。积极发展生态高效现代农业，提高农业绿色发展水平。优化消费环境，培育信息消费、环保消费和社区消费等新增长点，规划建设一批大型商业网点和精品专业市场，形成一批特色商业街区。

　　坚持强载体、兴产业、培育新增量。以10个园区和22个片区为载体，打造城市块状经济新高地。充分发挥高新技术开发区、综合保税区、明水经济技术开发区三个国家级园区示范引领作用，编制完成全市“产业地图”，以推进简政放权、完善基础设施、激活管理机制、壮大主导产业为重点，强化投资强度、产出效益等指标考核，力争10个省级以上园区生产总值、固定资产投资、财政收入、规模以上工业增加值均增长20%以上。按照连片开发、产城融合、职住平衡、中心复合的原则，探索实行区域综合审批等机制，强化片区差异化发展，统筹布局产业、居住、商业和基础设施，加快非遗园、鹊山龙湖、龙冈梦幻星空、绣源河、澄波湖、兴隆六个旅游发展区，西客站、奥体中心、华山、雪山、唐冶、彩石六个综合开发区，创新谷、济南药谷、汉峪金谷、中新科技城四个创新示范区，泺口、徐李、北湖、南北康四个改造提升区，新东站、火车站北场站两个交通枢纽区，共22个重点片区开发建设，统筹做好沿轨道交通、沿铁路、沿小清河两岸等新区域发展策划，妥善处理房地产与文化、旅游、商务等相关产业发展的关系，带动区域综合发展，打造可持续的产业、就业和税源。

　　（三）抢抓产业转型升级机遇，大力实施结构调整和创新驱动战略，塑造省会经济发展新优势

　　转型发展是我市产业做大做强的重大机遇。必须抢抓机遇，全力以赴打好转方式调结构攻坚战，做大做强优势产业，培育发展新兴产业，优化升级传统产业，抢占高端、创新驱动，努力赢得发展的战略主动，增强省会核心竞争力。

　　做大做强优势产业。对信息软件、交通装备、机械装备、食品药品、金融服务等我市五大优势产业，实施技术、产品、市场、组织和商业模式综合创新，拉长产业链，提高配套率，推动产业向价值链前后两端延伸。围绕做强信息软件产业，提升国家信息通信国际创新园、齐鲁软件园、量子通信研究院、齐鲁动漫基地等载体建设，实施更高水平中国软件名城建设计划，争创下一代互联网示范城市，构筑信息时代新优势。利用省会城市群经济圈城际铁路建设机遇发展轨道交通产业，依托重汽、吉利、青年、北车、轻骑、红帆等龙头企业，强化配套企业跟进，支持产业链缺项产品本地化生产，实现大交通装备产业集群发展。以高端智能装备为重点，依托二机床、铸锻所、济柴、齐鲁电机等企业，加快高端装备制造产业园、中电装备产业园等项目建设，在数控机床、电力设备、发动机、压力容器等方面突破核心技术，做大做强机械装备产业。依托齐鲁制药、福瑞达、福胶、旺旺、佳宝等骨干企业，加快国家重大新药研发大平台、齐鲁制药医药产业园、宏济堂中药产业园等平台建设，以生物制药、现代中药、医疗器械、医药物流、食品饮料为重点，做大规模、做响品牌，实现关键技术突破和产业化，壮大食品药品产业。推动金融改革创新，发展多层次资本市场，规范发展民间融资机构，发挥山东金融资产交易中心、路演中心的作用，鼓励企业抓住新三板扩容和上市重启机遇扩大融资，促进区域性金融中心建设。

　　加快发展新兴产业。立足产业链需求，促进战略性新兴产业和生产性服务业协同发展，打造高端高质高效产业体系。重点扶持新信息、新材料、新能源、生物医药等新兴产业和云计算、物联网、文化创意、研发设计、商务服务等生产性服务业。按照“专精特新”要求，培育晶正、蓝金、中商物联网、华凌电缆等一批科技含量高、成长前景好的“瞪羚企业”，壮大24个中小企业产业集群。突出抓好碳化硅半导体产业和光电子产业基地、德迈信息产业园等园区和项目建设。研究制定加快服务业发展的政策措施，强化规划、土地、资金等要素保障，支持在工业园区内按工业用地政策建设物流、研发、工业设计等配套服务业项目。大力发展总部经济，合理规划金融、信息技术、文化旅游、设计研发和综合型总部布局，抓大引强，吸引一批先进制造业、高新技术和现代服务业企业在济南集聚发展。

　　改造提升传统产业。强化市场主导，加快传统产业改造升级，提升产业素质和竞争优势。对钢铁、石化、纺织服装、建材等传统工业实施“六个一批”，加快转型升级，攻克一批关键技术，淘汰一批落后产能，做强一批新特优产品，壮大一批骨干企业，完善一批产业链条，培育一批产业集群，有序推进东部老工业区搬迁改造。创新住宿餐饮、批零贸易、物流会展等服务业经营模式，紧跟市场需求，加快提档升级，实施市场改造提升，建设医药、冷链、保税等7大物流产业集聚区及农产品物流中心等大宗商品交易平台，推进西区国际会展中心建设。

　　强化创新驱动发展。深入推进创新型城市建设，着重抓好平台建设、成果转化、完善机制、高端人才四个环节，支持鼓励各个领域、各类群体创业创新。依托53个国家级“金字招牌”助推创新发展，切实发挥创新谷、济南药谷、鑫茂科技城、航天科技城等孵化器、加速器在推动技术研发、成果转化、招商引资、产业培育、促进中小微企业成长等方面的作用，培育一批创新型产业集群。突出企业创新主体地位，健全技术创新市场导向机制，支持山东大学、省科学院等高校院所与企业协同创新，打造产业技术创新战略联盟。加强知识产权保护、运用和管理，深入实施专利、标准、品牌、商标战略，强化技术、市场、金融、法律等专业化配套服务，加快科技成果的产业化、规模化应用。把人才队伍和企业家队伍建设放在更突出的位置，建设“齐鲁人才特区”，培养引进一批领军人才、拔尖人才和紧缺适用人才，营造尊重企业家、支持企业家、尊重创业者、支持创新者的良好社会氛围。

　　（四）以新型城镇化为引领，提升城市功能品质，加快生态文明建设，推动美丽泉城建设取得重大进展

　　积极稳妥推进城镇化，构建以中心城区为核心、县域次中心城市为支撑、中心城镇和一般镇为基础、新型农村社区为补充的四级城镇化体系，进一步突出特色、优化空间、完善功能、创新管理，打造宜业宜居宜游省会新形象。

　　打造泉城特色。珍惜古城文化，突出泉水特色，完善历史文化名城保护规划，加快火车站北场站、明府城—百花洲片区、百年商埠等项目建设，分类整治老街巷，修缮题壁堂、老舍故居等历史建筑。改造提升千佛山、天下第一泉风景区等公园景区，加强对泉水强渗漏带的规划保护，办好第二届泉水节，扎实推进名泉申遗和“中国温泉之都”创建工作。

　　优化城区空间。坚持规划引领，按照生产空间集约高效、生活空间宜居方便、生态空间山清水秀的要求，以重点片区和城市功能性设施为支撑，加快“一城三区”和南部绿色发展、北部跨河发展等重点区域规划建设。加强地下空间开发利用，推进综合交通枢纽与城市公共中心的一体化发展，完善城中村改造体制机制政策，启动、续建92个旧村居改造，推动城市新区向新城转变。以生态旅游小镇建设为重点，推进南部山区交通、旅游和生态友好型农业发展，提高绿色发展水平。以产业北进带动北跨战略实施，完善体制机制和政策措施，实施长清黄河大桥等跨河通道和基础设施建设，加快新材料产业园、济北经济开发区、商河经济开发区建设，推进鹊山龙湖旅游度假区等重点项目取得实质性进展。

　　提升城市功能。编制综合交通体系规划，推进济青高铁、石济客专、新东站等项目，加快轨道交通R1线、二环南路高架及东延、二环东西高架路和顺河高架南延工程建设，推动凤凰路、纬十二路、济齐路等道路建设改造，完善城市快速路、主次干道网络，搞好支路建设，加大力度打通断头路，提升慢行步道质量水平。规划建设无轨电车BRT走廊，积极打造公交都市。实施“四供两排”管网建设改造，推进东区水厂、污水处理厂、第三生活垃圾处理厂建设，提高城市综合承载力。

　　创新城市管理。推进智慧泉城建设，搭建市、区、街道、社区四级城市管理物联网，对公共设施实行信息编码联网管理，创新整合城市管理运行机制，实现城市管理重心下移和城乡环卫一体化。深化环境综合整治，搞好城镇净化、绿化、亮化、美化，提高道路施工、管护、保洁水平，强力解决垃圾死角、渣土、扬尘、噪声污染等突出问题。加强建设工程监管，遏制新的违法建设产生。推进全领域安全生产大检查，消除地下油气水电热等隐蔽管线致灾隐患，加强交通、排水、消防安全管理，确保城市安全运行。

　　创建生态济南。启动全市生态环境总体规划编制，对南部山区、沿黄湿地、城市水系、公共绿地等重大生态功能区域实行严格的生态保护。调整能源结构，促进工业节能、建筑节能、交通节能，发展风力发电等新能源产业，实施废热利用和热电联供工程，城市建成区内禁止新上燃煤锅炉，力争2015年实现煤炭消费总量“不增反降”。淘汰钢铁、水泥、化工等行业落后产能，严厉查处偷排偷放、超标排放等违法行为，确保污染物达标排放。发展绿色建筑，推进太阳能光热、光电建筑一体化应用，从今年起，100米以下新建高层住宅和集中使用热水的公共建筑全部推广使用太阳能。强化雾霾天气综合治理，建立重污染天气预警机制和空气质量保障应急预案制度，妥善处置环境公共安全事件。加强机动车环保管理，2015年底前淘汰全部黄标车。统筹推进水生态文明市创建等“五城联创”工作，实施玉符河、兴济河等河道综合治理，启动华山湖湿地建设和大明湖小清河连通工程，开展水系、荒山、道路、社区、村庄以及裸露土地绿化，开工建设北绕城高速、北大沙河等生态隔离带和防护林带，提升济青、济菏高速等绿色通道绿化水平，新增造林面积20万亩，加快长平滩区护城堤建设。推进农村人居环境整治，注重保留特色村庄传统风貌，打造一批富裕、宜居、秀美、和谐、活力的美丽乡村。

　　（五）进一步突出农业基础地位，立足特色优势，强化产业支撑，尽快实现县域经济突破性发展

　　把做大做强县域经济作为富民强市的一个战略基点，统筹抓好粮食生产、产业培育、基础设施建设和公共服务保障，实现农业增产、农民增收、农村增彩，激发县域发展的内生动力。

　　确保粮食安全。坚守基本农田不减少、粮油生产能力不降低、巩固农民持续增收机制三条底线，守住全市549万亩耕地红线。推进粮食增产工程，抓好120万亩粮食生产功能区建设，尽快在全市恢复10万亩水稻种植，加强小农水重点县、“五小”水利等基础设施建设，实施15项农业科技创新项目，推广农业机械化和良种良法生产配套技术，提高农业生产率。实施农产品全程质量安全管理，严格管制乱用、滥用农业投入品，加快农产品质量标准、检测和认证三大体系建设，从源头上确保广大人民群众“舌尖上的安全”。

　　加快发展现代农业。健全农业产业化体系，发展设施农业、循环农业、高端品牌农业和现代都市农业，抓好蔬菜、畜牧、林果、花卉苗木四大主导产业和“一区六园”建设，做优做强前端现代种业、后端农产品销售配送和深加工。加快构建新型农业经营体系，壮大新型农业经营主体，重点培育30家农业龙头企业、30家合作社示范社、30家农业示范园区、30家示范性家庭农场和30家市级畜禽标准化养殖场，鼓励发展“公司+基地+农户”合作模式，提高集约经营、规模经营、社会化服务水平。稳定农村土地承包权，积极推进土地承包经营权确权登记颁证试点，推动土地经营权流转，开展土地承包经营权抵押贷款试点，探索建立综合性农村产权交易市场和城乡建设用地增减挂钩节余指标市场交易机制。积极开展联保质押、银企对接等服务，推动齐鲁银行网点快速向全省发展，争取年内组建济南农村商业银行。开辟新型农民教育培训基地，实施农村劳动力培训阳光工程。探索整村推进和精准化扶贫相结合的产业扶贫开发新模式，加快6万低收入农民增收脱贫步伐。

　　做强县域特色优势产业。依托炭素、玛钢、树脂、食品饮料、液压升降机、压力容器、纺织服装等县域优势产业以及特色农产品、独特自然和历史文化资源，坚持三次产业协同发展，打通产业间隔，延伸产业链条，提高本地原料配套供给比重，带动上下游产业融合发展。大力发展金融、物流、信息等生产性服务业，引导资金、产业、人才集聚园区，提高县域产业集群集约发展水平。培育一批龙头骨干企业，支持企业开拓市场、强化管理、研发创新、跨业经营，实现就地扩规提效、增加就业、膨胀发展。因地制宜发展特色镇域经济，宜工则工、宜商则商、宜游则游，加快推动以11个省级示范镇、3个市级示范镇为重点的小城镇建设，打造一批工业强镇、商贸大镇、旅游名镇，将镇域建设成吸纳农村劳动力的蓄水池、产品流通的周转站、产业发展的集聚地，增强县域经济竞争力。

　　（六）创新机制、搭建平台，着力提升集聚辐射能力，构筑对内对外开放发展新格局

　　不断提高对外开放水平，构建对内和对外开放相互促进、国际国内市场深度融合的全方位开放型经济新体系。

　　打造对外开放高端平台。推进新加坡（济南）国际科技城规划建设，以现代服务业和高端制造业为主导，打造具有国际示范意义、引领开放经济发展的科技新城。加快济南航空城规划建设。探索建设鲁台港澳金融合作示范区，积极推进花旗银行等外资金融机构入驻济南。实施海关与检验检疫 “一次申报、一次查验、一次放行”通关模式试点，争创跨境贸易电子商务服务试点城市。加快建设国际学校、国际医院。

促进外贸转型发展。完善综合保税区运行机制，加快建设保税功能拓展区和配套产业区，推动组装加工向研发、设计、核心元器件制造、现代物流等环节拓展，实现加工贸易扩规转型。以齐鲁外包城为载体，带动新一轮服务外包产业发展。保持大型成套装备、汽车、管件等传统出口优势，创造新的比较优势和竞争优势，扩大我市转型升级所需设备和技术进口。积极融入中日韩自由贸易区建设发展。

加快“海外济南”建设。强化对外推介宣传，对“走出去”企业加强宏观指导和服务。支持重卡、太阳能、铁矿石等八个海外产业和资源基地建设。推进国际营销网络建设，深度拓展非洲市场，在俄罗斯、东盟等地区探索设立济南产品贸易、展示、仓储中心，鼓励企业赴境外建立地区销售服务总部、海外专卖店、加工配送中心或授权境外特许经营。发挥外事、侨务、对台、贸促等纽带作用，推进友城经贸合作，探索建立产业合作示范城市。

　　推动省会城市群经济圈一体化发展。按照“五个同城化”要求，率先在济莱协作区实施突破。组建钢铁、装备、软件、旅游等产业联盟，促进产业优势互补、协作融合和战略转移。整合推出泰山、天下第一泉等一体化旅游线路，打造整体旅游品牌。推动基础设施共建共享、软硬件系统互联互通。加快济泰、济聊高等级公路和济莱快速通道，济南至乐陵、济南至东营高速公路建设，重点推进济莱、济聊城际铁路建设。发挥“一小时生活圈”功能，推行居民日常生活“一卡通”，整合高端商业资源。完善联席会议制度和区域协作长效机制，促进区域融合发展。

　　（七）稳步提高民生保障水平，创新社会治理，进一步促进社会公平正义与和谐稳定

　　按照中央提出的“守住底线、突出重点、完善制度、引导舆论”的思路，积极做好改善民生各项工作，在创新治理中加强社会建设，不断提高人民群众生活质量。

　　大力鼓励创新创业。促进以高校毕业生为重点的青年就业，统筹做好农村转移劳动力、城镇困难人员、退役军人就业。提升“阳光大姐”品牌，促进妇女就业，推动家政服务业发展。鼓励创业促就业，落实小额贷款等扶持政策，建设一批创业孵化基地和园区。确保完成新增城镇就业10 万人、农村劳动力转移就业5.2万人，援助就业困难人员2万人，高校毕业生总体就业率稳定在80%以上。

　　深化基础保障改革。按照保住基本、补上短板、兜好底线的要求，继续扩大养老、医疗、失业、生育、低保、救助等基础保障覆盖面，逐步提高保障标准。推动养老事业与养老产业互动发展，加快市养老服务中心等养老机构和服务信息平台建设。推进保障性住房建设，向符合条件的申请家庭发放廉租住房租金补贴。

　　加快文化教育事业发展。统筹教育领域综合改革，合理调整学校规划布局，加快教育均衡发展。围绕产业发展和就业需求发展职业教育，培养更多高素质技能人才。做好外来务工人员子女入学工作。在市、县区公共图书馆、文化馆、艺术馆、博物馆等普遍建立理事会，发挥省会文化艺术中心等公共文化场馆作用，做好基层公共文化设施建设、文化信息资源共享等文化惠民工程，继续推进百所博物馆建设，实施文化和自然遗产保护工程，承办好第三届中国非物质文化遗产博览会。

　　深化医药卫生体制改革。加快公立医院改革，逐步取消以药补医。健全重、特大疾病保障机制。实施机关事业单位公费医疗改革。做好济阳、商河县级公立医院综合改革试点，推进医药价格和医保支付方式改革。鼓励社会力量开办医疗机构或参与公立医院改制重组。

　　创新社会治理。推进城乡社区网格化服务管理。培育协会、商会等社会组织和专业社工、志愿者队伍，实现政府治理、社会调节和居民自治有机结合。创新预防化解社会矛盾体制，以制定国家标准为契机提升12345市民服务热线工作水平，通过网上受理信访、复议等形式，及时解决群众合理诉求。坚持计划生育基本国策，落实完善生育政策。深化户籍制度改革，有序推进农业转移人口市民化。加大社会治安综合治理，开展普法宣传，建设“平安济南、法治济南”。加大工作力度，全力争创全国文明城市。完善监管机构，深化专项整治，切实保障食品药品安全。进一步做好双拥共建、人民防空、民兵预备役工作，推动军民融合发展。精心组织第三次经济普查工作。继续做好民族宗教、社会科学、老龄、慈善、红十字、残疾人等工作。

　　谋民生之利，解群众之忧，是政府职责的根本所在。今年将围绕城乡环卫一体化、废热利用、绿色建筑、农村义务教育、保障性住房、养老服务、基层群众文化活动、农民工服务、法律援助等方面，继续办好保障和改善民生十五件实事。在此基础上，我们将继续倾听广大群众的诉求和呼声，多做雪中送炭的事情，实事求是解难题、纾民忧、办实事，扎实有序做好保基本的工作，努力让发展成果更好地惠及民生。

　　各位代表，党的十八届三中全会吹响了新一轮改革开放的号角，全市深化改革、科学发展、保障民生、维护稳定的任务十分艰巨，这对加强政府自身建设提出了更高要求，我们深感责任重大。空谈误国，实干兴邦，再美好的规划，再宏伟的蓝图，归根到底要靠真抓实干才能实现。我们将深入贯彻落实党的十八大和十八届三中全会精神，践行党的群众路线，把握我市发展新要求和人民群众新期待，解放思想、提升境界，围绕大局、服务发展，勇于担当、积极作为，努力提高政府治理能力，促进政府效率提升，推动政府各项工作开创新局面。我们将把狠抓落实摆在突出位置，全面提高公务员队伍素质，加强学习、提高标准、开拓创新、攻坚克难，力戒形式主义、官僚主义、享乐主义和奢靡之风，及时解决改革发展的重点难点问题，推动落实好“加快科学发展，建设美丽泉城”各项工作。我们将严格执行《党政机关厉行节约反对浪费条例》，加强反腐败体制机制创新和制度保障，积极推进公车改革、办事公开、部门述职等制度建设，推进财政预决算和“三公经费”、环境保护、安全生产等社会关切事项公开，开辟和畅通政府网站等依申请公开办理渠道，加快建设网上政府。我们将认真接受市人大及其常委会法律监督、工作监督和市政协民主监督，主动听取各民主党派、工商联、各人民团体和各界人士意见建议，加大对议案、提案和意见建议办理落实力度。我们将坚持运用法治思维和法治方式深化改革、推动发展、化解矛盾，严格按法定权限和程序行使权力，切实用制度管权管事管人，让权力在阳光下运行，努力建设法治政府和服务型政府。

　　各位代表，新的征程已经开启，科学发展重任在肩。让我们高举中国特色社会主义伟大旗帜，紧密团结在以习近平同志为总书记的党中央周围，认真贯彻落实党的十八大和十八届三中全会精神，在省委省政府和市委坚强领导下，锐意进取，真抓实干，共同创造济南更加美好的未来！
